# EPSTI1 Is Involved in IL-28A-Mediated Inhibition of HCV Infection

**DOI:** 10.1155/2015/716315

**Published:** 2015-06-03

**Authors:** Xianghe Meng, Darong Yang, Rong Yu, Haizhen Zhu

**Affiliations:** ^1^Department of Molecular Medicine of College of Biology, State Key Laboratory of Chemo/Biosensing and Chemometrics, Hunan University, Changsha 410082, China; ^2^Research Center of Cancer Prevention & Treatment, Translational Medicine Research Center of Liver Cancer, Hunan Provincial Tumor Hospital, Affiliated Tumor Hospital of Xiangya Medical School of Central South University, Changsha 410013, China

## Abstract

It has been reported that IFN-*λ*s inhibit HCV replication in vitro. But the mechanisms of how IL-28A conducts antiviral activity and the functions of IL-28A-induced ISGs (IFN-stimulated genes) are not fully understood. In this study, we found that IL-28A has the antiviral effect on HCV life cycle including viral replication, assembly, and release. IL-28A and IFN-*α* synergistically inhibit virus replication. EPSTI1 (epithelial-stromal interaction 1), one of IL-28A-induced ISGs, plays a vital role in IL-28A-mediated antiviral activity. Furthermore, forced expression of EPSTI1 effectively inhibits HCV replication in the absence of interferon treatment, and knockdown of EPSTI1 contributes to viral enhancement. EPSTI1 can activate PKR promoter and induce several PKR-dependent genes, including IFN-*β*, IFIT1, OAS1, and RNase L, which is responsible for EPSTI1-mediated antiviral activity.

## 1. Introduction

Hepatitis C virus (HCV) is a major public health problem that infects approximately 170 million people worldwide [1]. No vaccine is available for prevention of HCV infection. As a member of the Flaviviridae, HCV's positive-sense RNA genome contains highly structured 5′ and 3′ nontranslated regions (NTRs) flanking a large open reading frame encoding a polyprotein including structural (core-E2) and nonstructural (NS) proteins. Alpha interferon- (IFN-*α*-) based therapy, a regimen for HCV, is effective at best in only 50% of patients [2]. Although the protease inhibitors that target HCV NS3/4A have been licensed to treat chronic HCV infection [3], many patients may be resistant to the therapy because of viral genome mutation in the presence of the protease inhibitor or even before the treatment [4, 5].

In the past years, genetic studies have identified several single nucleotide polymorphisms (SNPs) (rs12979860, rs12980275, and rs8099917) around the IL-28B (interleukin-28B and interferon *λ*3) gene, which are strongly associated with sustained viral response to PEG-IFN and RBV treatment for chronic hepatitis C [6–8]. IL-28B haplotypes are also strongly associated with spontaneous HCV clearance [6, 9, 10]. IFN-*λ*s are categorized as type III IFNs, including IL-29 (IFN-*λ*1), IL-28A (IFN-*λ*2), and IL-28B (IFN-*λ*3), which are potent and endogenous antiviral cytokines. Although their structures are very homologous to IL10 family members, IFN-*λ*s are more functionally similar to type I IFNs (IFN-*α*/-*β*) [11]. They signal via intracellular Jak/Stat pathways and induce transcription of IFN-stimulated genes (ISGs) for control of viral infection. It was reported that IFN-*λ*s inhibit HCV replication in vitro [12–15]. The clinical trials of IFN-*λ*1 in patients with chronic HCV infection have shown promising results, which indicated that 86% of patients who received combined pegylated (PEG) IFN-*λ* and ribavirin (RBV) for 4 weeks had a more than 2log_10_ IU/mL decrease in HCV RNA [16].

The development of infectious HCV clone (JFH1) provides a powerful tool for investigation of antiviral drugs [17, 18]. The mechanisms of how IL-28A conducts antiviral activity and the functions of IL-28A-induced ISGs are not fully understood. In this study, we used infectious cell culture system to explore the antiviral effect of IL-28A on HCV life cycle. Our data showed that IL-28A inhibits HCV replication, assembly, and release without affecting HCV entry. IL-28A and IFN-*α* synergistically suppress HCV replication. The peak ISGs expression induced by IFN-*α* occurs earlier than that induced by IL-28A, and the combination of IL-28A and IFN-*α* synergistically upregulates the expression of ISGs. EPSTI1 (epithelial-stromal interaction 1) is upregulated in tissues characterized by extensive epithelial-stromal interaction, which promotes invasion and metastasis of breast cancer [19]. EPSTI1 can replace peritumoral activated fibroblasts in the tumor microenvironment [20]. However, the potential function of EPSTI1 to restrict viral infection is unknown. In this study, we found that EPSTI1, one of IL-28A-induced ISGs, effectively inhibits HCV replication and plays an essential role in IL-28A-mediated anti-HCV activity. Furthermore, the antiviral activity of EPSTI1 is dependent on PKR pathway.

## 2. Materials and Methods

### 2.1. Cell Culture and Reagents

FL-neo, a HCV 1b full-length replicon cell line, and Huh7.5 cells were kindly provided by Dr. Charles M. Rice (Rockefeller University, New York, NY). HLCZ01 cells were established in our lab [21]. All cells were propagated in Dulbecco's modified Eagle medium (DMEM) supplemented with 10% FBS, L-glutamine, nonessential amino acid, penicillin, and streptomycin. Recombinant human IL-28A and IFN-*α* were purchased from R&D Systems.

### 2.2. HCV Constructs and Viral Particle Generation

pJFH-1 and pJFH-1/GND plasmids were gifts from Dr. Takaji Wakita (National Institute of Infectious Diseases, Tokyo, Japan) [17]. The linearized DNA was purified and used as a template for in vitro transcription using MEGAscript kit (Ambion, Austin, TX). In vitro transcribed genomic JFH-1 or JFH-1/GND RNA was delivered into Huh7.5 cells by electroporation. The transfected cells were transferred to complete DMEM and cultured for the indicated period. Cells were passaged every 3–5 days, and corresponding supernatants were collected and filtered with a 0.45 *μ*m filter device. The viral titers were expressed as focus-forming units (FFU) per milliliter, determined by the average number of NS5A-positive foci detected in Huh7.5 cells [18].

### 2.3. Intracellular Virus Isolation

At the indicated time after HCV infection, cells were washed thrice with PBS and incubated with 0.25% of trypsin-EDTA for 2 min at 37°C. Cells were suspended in PBS and collected by centrifugation at 2,000 rpm for 3 min. The cell pellet was resuspended in Dulbecco's modified Eagle medium- (DMEM-) 10% fetal bovine serum (FBS) and lysed by four freeze-thaw cycles in liquid nitrogen and a 37°C water bath, respectively. Cell debris was collected by centrifugation at 4,000 rpm for 5 min. The supernatant was reserved and used for the focus-forming units (FFU) assay to determine viral titers.

### 2.4. Real-Time PCR Assay

Total cellular RNA was extracted using TRIzol (Invitrogen, Carlsbad, CA) according to the manufacturer's protocol. Superscript III First-Strand Synthesis Kit for reverse transcription of RNA was purchased from Invitrogen. The primers that targeted HCV, GAPDH, and 1-8U have been reported and real-time PCR was performed as described previously [22].

### 2.5. Plasmid Construction

EPSTI1 cDNA was amplified from total cellular RNA isolated from Huh7.5 cells using standard RT-PCR and subsequently subcloned into the p3×FLAG-CMV. The primers for amplification of EPSTI1 are 5′-GGAATTCCATGAACACCCGCAATAGAGT-3′ (forward EcoR I) and 5′-GCTCTAGAGCAGAAAAATAATGTAGCATTTCCC-3′ (reverse Xba I). The shRNAs targeting EPSTI1 were constructed into pSilencer-neo plasmid (Ambion). The sequences of EPSTI1 shRNAs targeting three regions of EPSTI1 were 5′-AACAACAACTCCAGCTGATGC-3′, 5′-AACCGCTGAGTTCTTGAGCAA-3′, and 5′-AAGATGAAGGATGAACAACAT-3′. The negative control shRNA plasmid was purchased from Ambion.

### 2.6. Antibodies

Monoclonal antibodies against actin, EPSTI1, STAT1, STAT3, phosphorylated STAT1, and phosphorylated STAT3 were obtained from Santa Cruz Biotechnology. Monoclonal antibodies against PKR and phosphorylated PKR were from Sangon Biotech. Mouse monoclonal anti-NS5A antibody was a gift from Dr. Chen Liu (University of Florida, Gainesville, FL). Goat anti-mouse and goat anti-rabbit IgG-HRP secondary antibodies were purchased from Santa Cruz Biotechnology.

### 2.7. Western Blot Analysis

The procedure was reported previously [23]. Briefly, cells were washed with PBS and lysed in RIPA buffer (150 mM sodium chloride, 1% Nonider P-40, 0.5% sodium deoxycholate, 0.1% SDS and 50 mM Tris-HCl (pH 8.0) supplemented with 2 *μ*g/mL of aprotinin, 2 *μ*g/mL of leupeptin, 20 *μ*g/mL of phenylmethylsulfonyl fluoride, and 2 mM DTT). Forty micrograms of protein was resolved by SDS/PAGE, transferred to PVDF membrane, and probed with appropriate primary and secondary antibodies. The bound antibodies were detected by Supersignal West Pico Chemiluminescent Substrate (Pierce, Rockford, IL).

### 2.8. Immunofluorescence

The protocol has been described previously [24].

### 2.9. Statistical Analysis

Student's *t*-test was applied to determine statistical significance. Bars represented S.D. ^*∗*^ *P* < 0.05, ^*∗∗*^ *P* < 0.01, and ^*∗∗∗*^ *P* < 0.001 versus control.

## 3. Results

### 3.1. IL-28A Suppresses HCV Replication

**Figure 1:**
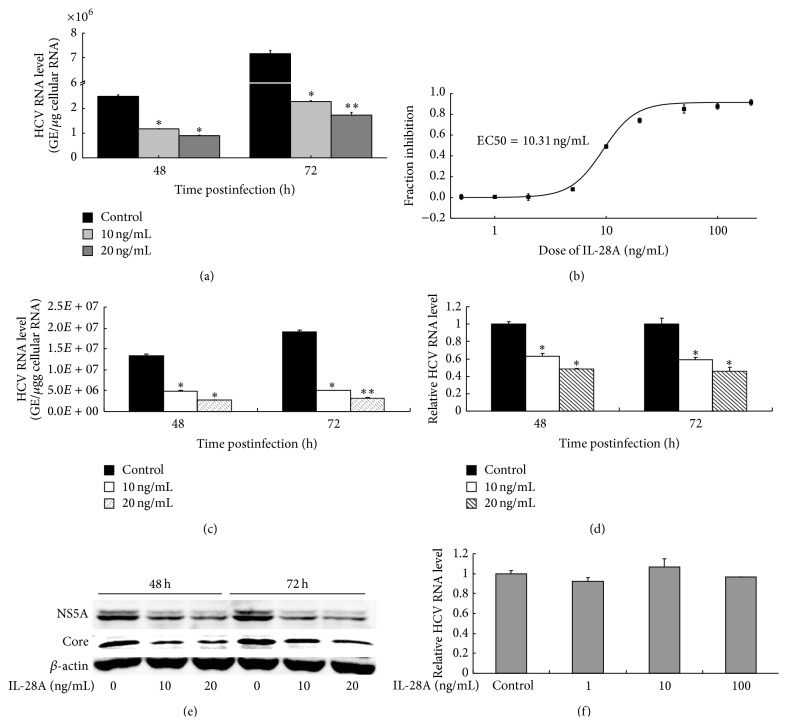
IL-28A suppresses HCV replication. Huh7.5 (a) and HLCZ01 (c) cells were infected with HCV (MOI = 0.1) for 6 h at 37°C. The medium was then removed and cells were incubated with IL-28A for 48 or 72 h at 37°C. (b) HCV-infected Huh7.5 cells were incubated with different doses of IL-28A for 72 h. (d) FL cells were incubated with IL-28A for 48 or 72 h. ((a), (b), (c), and (d)) Intracellular viral RNA was detected by real-time PCR. The results are the average of three independent experiments performed in triplicate. (e) Huh7.5 cells were treated as described in part (a). NS5A and core proteins were detected with western blot. (f) Huh7.5 cells were pretreated with or without IL-28A for 12 h. The medium was then removed, and cells were inoculated with HCV (MOI = 0.1) for 6 h at 37°C. Intracellular viral RNA was detected by real-time PCR.

In previous study, we have investigated the anti-HCV activity of IL-28A in HCV subgenomic replicon cell line [12]. The recent development of infectious HCV cell culture system, a genotype 2a patient isolate JFH1, facilitates the study of anti-HCV drugs. Here, we assessed the antiviral activity of IL-28A in infectious cell culture system and a HCV genotype 1b full-length replicon cell line (FL-neo). Huh7.5 cells were inoculated with purified JFH1 at MOI of 0.1 for 6 hours. After the removal of inoculum, the cells were cultured with the fresh media containing different doses of IL-28A. Viral RNA replication in Huh7.5 cells was suppressed by IL-28A in both dose- and time-dependent manners (Figure 1(a)). The EC50 of IL-28A for inhibition of HCV RNA replication in Huh7.5 cells was 10.31 ng/mL (Figure 1(b)). We also assessed the antiviral activity of IL-28A in our newly developed hepatoma cell line HLCZ01 with HCV infection [24]. IL-28A inhibited HCV RNA replication in viral-infected HLCZ01 cells in both dose- and time-dependent manners (Figure 1(c)). Moreover, IL-28A also repressed HCV RNA replication in FL-neo cells (Figure 1(d)), suggesting that IL-28A may act as an antiviral drug for multigenotypes of HCV. To further confirm the inhibition of IL-28A on HCV replication, we detected viral proteins in the cells. As shown in Figure 1(e), NS5A and core proteins were significantly reduced in HCV-infected cells with IL-28A treatment. To investigate whether IL-28A has effect on HCV entry, we pretreated Huh7.5 cells with IL-28A for 12 hours followed by HCV infection for 6 hours. The result indicated that IL-28A treatment does not affect the efficiency of HCV entry (Figure 1(f)). These data suggested that IL-28A inhibits HCV replication without affecting HCV entry.

### 3.2. IL-28A Inhibits Assembly and Release of Infectious HCV Particles

**Figure 2:**
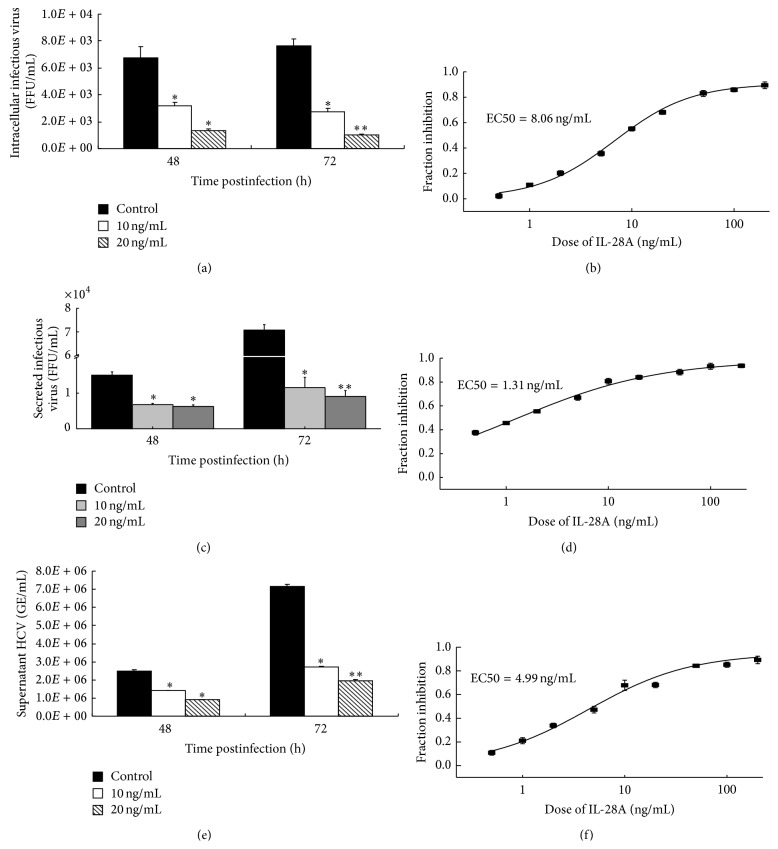
IL-28A inhibits assembly and release of infectious HCV particles. ((a) and (c)) Huh7.5 cells were infected with HCV (MOI = 0.1) for 6 h at 37°C. The medium was then removed and cells were incubated with IL-28A for 48 or 72 h at 37°C. The intracellular virus particles (a) and the cell culture supernatants (c) were harvested and titered by FFU (focus-forming unit) assay on naive Huh7.5 cells. (e) HCV RNA in the cell culture supernatants was extracted and detected with real-time PCR. ((b), (d), and (f)) The dose-response curves of IL-28A. (b) The intracellular virus particles were detected as described in part (a). (d) The secreted infectious virus particles were detected as described in part (c). (f) HCV genome equivalents were detected as described in part (e). The results are the average of three independent experiments performed in triplicate.

The processes of viral assembly and release are essential for virus production. To determine whether IL-28A has effect on these two steps of HCV life cycle, we detected the infectious viral particles inside HCV-infected cells and in the supernatant of cell culture. The infectious HCV particles in IL-28A-treated Huh7.5 cells (Figure 2(a)) as well as in the supernatant of the cells with IL-28A treatment (Figure 2(c)) were significantly decreased in comparison with mock-treated control. The EC50 of IL-28A to suppress the intracellular viral assembly and extracellular viral release were 8.06 ng/mL (Figure 2(b)) and 1.31 ng/mL (Figure 2(d)), respectively. We also investigated the level of secreted HCV RNA in the supernatant of IL-28A-treated cells. Consistent with the result of focus-forming unit (FFU) assay, secreted HCV RNA was markedly reduced in the supernatant of IL-28A-treated cells (Figure 2(e)) and the EC50 was 4.99 ng/mL (Figure 2(f)).

The decrease of the intracellular and extracellular infectious particles and the reduction of secreted HCV RNA may be caused by inhibition of viral RNA replication by IL-28A. To confirm the inhibitory effect of IL-28A on HCV assembly and release, we detailedly analyzed the percentages of the reduction of intracellular viral RNA, intracellular infectious particles, extracellular viral RNA, and extracellular infectious particles. Interestingly, the inhibitory percentages of intracellular infectious particles at both 48 h and 72 h were markedly higher than that of intracellular HCV RNA, indicating that IL-28A inhibits viral assembly (see Figure S1 in the Supplementary Material available online at http://dx.doi.org/10.1155/2015/716315). On the other hand, the inhibitory percentages of extracellular HCV RNA at both time points were also larger than that of intracellular HCV RNA, suggesting that IL-28A blocks viral release (Figure S1). These results demonstrated that IL-28A exhibits antiviral effect on both virus assembly and release in vitro.

### 3.3. IL-28A and IFN-*α* Combination Synergistically Inhibits HCV Replication and Induces ISGs Expression

**Figure 3:**
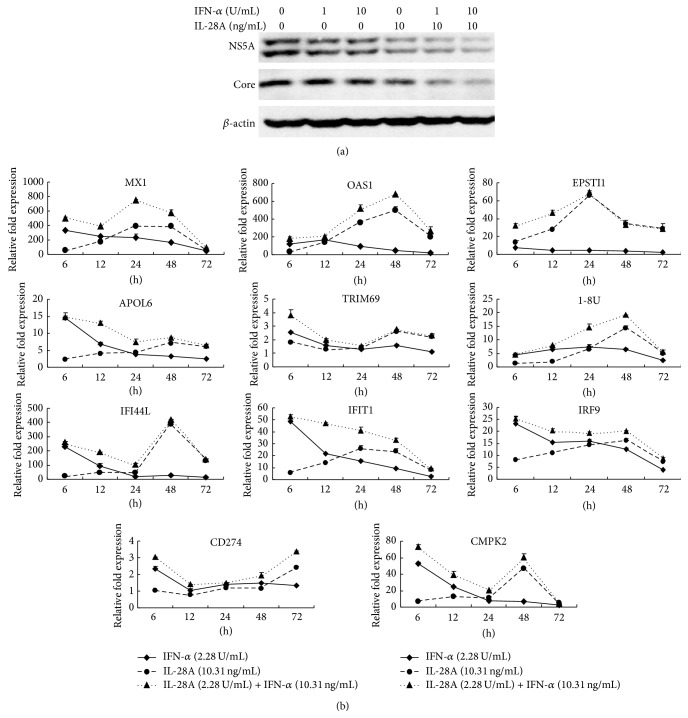
IL-28A and IFN-*α* combination synergistically inhibits HCV replication and induces ISGs expression. (a) Huh7.5 cells were infected with HCV (MOI = 0.1) for 6 h at 37°C. The medium was then removed and cells were incubated with IL-28A or IFN-*α* or together for 48 h at 37°C. HCV NS5A and core proteins were detected by western bolt. (b) Huh7.5 cells were treated by IL-28A or/and IFN-*α* for different time courses. The expressions of ISGs, including MX1, OAS1, EPSTI1, APOL6, TRIM69, 1-8U, IFI44L, IFIT1, IRF9, CD274, and CMPK2, were determined by real-time PCR and normalized with GAPDH.

Our previous study showed that IL-28A can signal through the JAK-STAT pathway in a similar manner as IFN-*α* [12]. To determine whether IL-28A enhances IFN-*α*-induced anti-HCV activity, we treated HCV-infected Huh7.5 cells with IFN-*α* alone or combination with IL-28A. As shown in Figure 3(a), the reduction of HCV NS5A and core proteins with combination of IL-28A and IFN-*α* treatment was more remarkable than that treated with IFN-*α* or IL-28A alone at 48 hours. These data indicated that the combination of IL-28A and IFN-*α* has a synergistic anti-HCV effect.

Type I (IFN-*α*/-*β*), type II IFN (IFN-*γ*), and type III (IL-28A, IL28B, and IL29) have well-described antiviral properties [25]. Each kind of IFNs induces a unique and partially overlapping set of ISGs [26–28]. The products of these ISGs exert numerous antiviral effector functions, however, many of which are not fully described [29]. To compare the different effects of ISGs induction between IL-28A and IFN-*α*, the EC50 doses of IL-28A (10.31 ng/mL) and IFN-*α* (2.02 U/mL) to inhibit HCV RNA replication were chosen for subsequent experiments (Figures 1(b) and S2). In our study, we detected a series of ISGs induced by IL-28A and IFN-*α* alone or in combination using real-time RT-PCR. Eleven ISGs, including Mx1, OAS1, EPSTI1, APOL6, TRIM69, 1-8U, IFIT1, IFI44L, IRF9, CD274, and CMPK2, presented as the time-dependent expressional changes. Most of these genes induced by IFN-*α* peaked before 12 hours and fell thereafter (Figure 3(b)). In contrast, the expression of IL-28A-upregulated genes peaked between 24 and 48 hours and fell at 72 hours except CD274 which was continuously induced up to 72 hours (Figure 3(b)). The levels of induced ISGs with dual treatment of IFN-*α* and IL-28A were higher than that treated with IFN-*α* or IL-28A individually, especially at early time course (Figure 3(b)). These results suggested that the ISGs induced by IFN-*α* occur earlier than that induced by IL-28A, and the combination of IL-28A and IFN-*α* synergistically upregulates ISGs expression.

### 3.4. EPSTI1 Exhibits Anti-HCV Activity

**Figure 4:**
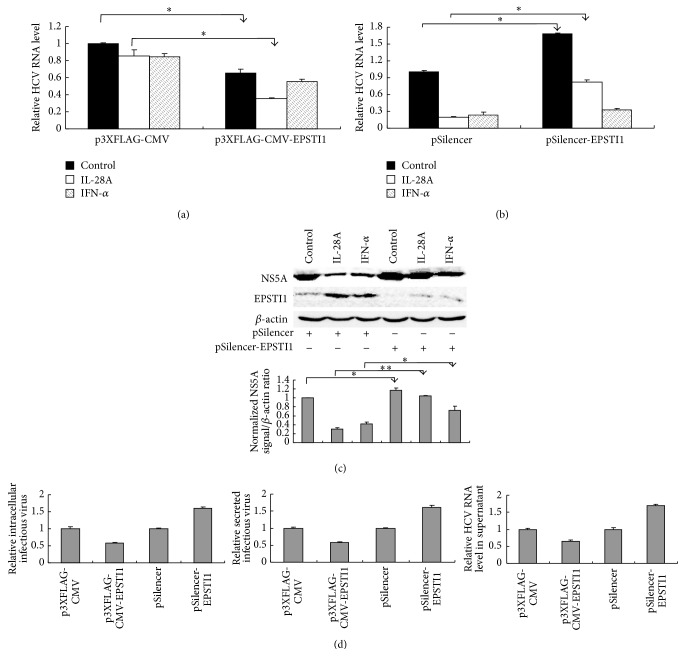
EPSTI1 exhibits anti-HCV activity. (a) Huh7.5 cells were transfected with p3XFLAG-CMV-EPSTI1 or vector followed by HCV infection (MOI = 0.1). The cells were incubated with IL-28A (5 ng/mL), IFN-*α* (0.5 U/mL) for 48 h, or without treatment as a control. Intracellular HCV RNA was detected with real-time PCR and normalized with GAPDH. (b) EPSTI1 shRNA or control shRNA was delivered into Huh7.5 cells. The cells were infected with HCV (MOI = 0.1) and incubated with IL-28A (20 ng/mL), IFN-*α* (5U/mL) for 48 h, or without treatment as a control. (c) The cells were treated as described in part (b). The proteins of NS5A and EPSTI1 were detected with western bolt. (d) Huh7.5 cells were transfected with p3XFLAG-CMV-EPSTI1/pSilencer-EPSTI1 or vectors followed by HCV infection (MOI = 0.1). The intracellular infectious virus and the secreted infectious virus were detected with FFU assay. HCV RNA in the supernatant was extracted and detected with real-time PCR.

The majority of ISGs selected in this study have been identified as anti-HCV genes [29]. However, the function of EPSTI1 is unclear and whether EPSTI1 also has antiviral activity is unknown. Moreover, EPSTI1 was upregulated more markedly by IL-28A than IFN-*α* treatment (Figure 3(b)). Hence, we hypothesized that EPSTI1 may play a pivotal role in IL-28A-mediated anti-HCV activity. To explore the function of EPSTI1, we conducted a series of experiments using gain- or loss-function strategies. Overexpression of EPSTI1 inhibited HCV RNA replication and enhanced the inhibitory effects of IL-28A on viral RNA replication (Figure 4(a)). On the other hand, silencing of EPSTI1 by shRNA facilitated HCV RNA replication and harmed the repression of viral replication by IL-28A (Figure 4(b)). In addition, the reduction of HCV NS5A protein triggered by IL-28A was markedly reversed by knockdown of EPSTI1. Although silencing of EPSTI1 also slightly attenuated the anti-HCV activity of IFN-*α*, the significance of this change by IL-28A was more remarkable. The effects of EPSTI1 silencing by shRNA and EPSTI1 induction by each interferon were verified by western blot (Figure 4(c)). These data suggested that EPSTI1 inhibits HCV replication and plays an important role in IL-28A-induced viral clearance.

Next, we tested the impact of EPSTI1 on other steps of HCV life cycle. Interestingly, the production of intracellular and extracellular infectious HCV particles and the secretion of HCV RNA were affected by overexpression or knockdown of EPSTI1 (Figure 4(d)). However, this may be caused by EPSTI1-triggered affection of viral RNA replication, because the change folds of intracellular and extracellular infectious HCV particles production and HCV RNA release were nearly similar to that of viral RNA replication (Figures 4(a), 4(b), and 4(d)). Therefore, we concluded that EPSTI1 may have no additional inhibition on HCV assembly and release.

### 3.5. The Inhibition of HCV Triggered by EPSTI1 Is Dependent on PKR

**Figure 5:**
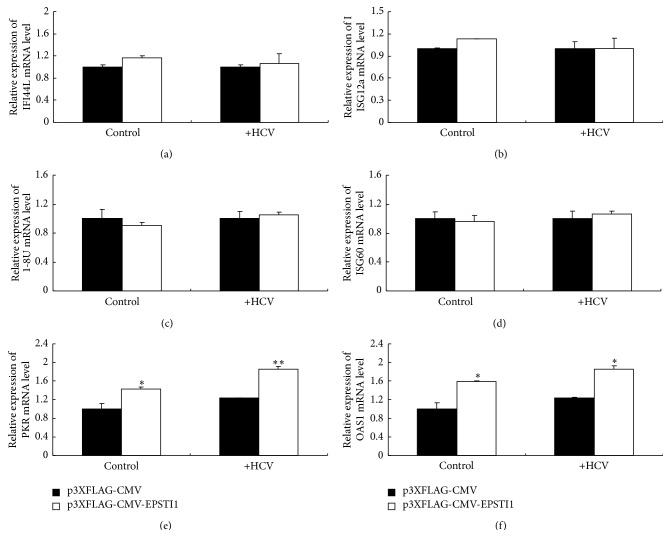
The regulation of ISGs by EPSTI1. Huh7.5 cells were transfected with p3XFLAG-CMV-EPSTI1 or vector followed by HCV infection (MOI = 0.1). The cells were harvested at 48 h after infection. IFI44L (a), ISG12a (b), 1-8U (c), ISG60 (d), PKR (e), and OAS1 (f) mRNA were detected by real-time PCR and normalized with GAPDH.

**Figure 6:**
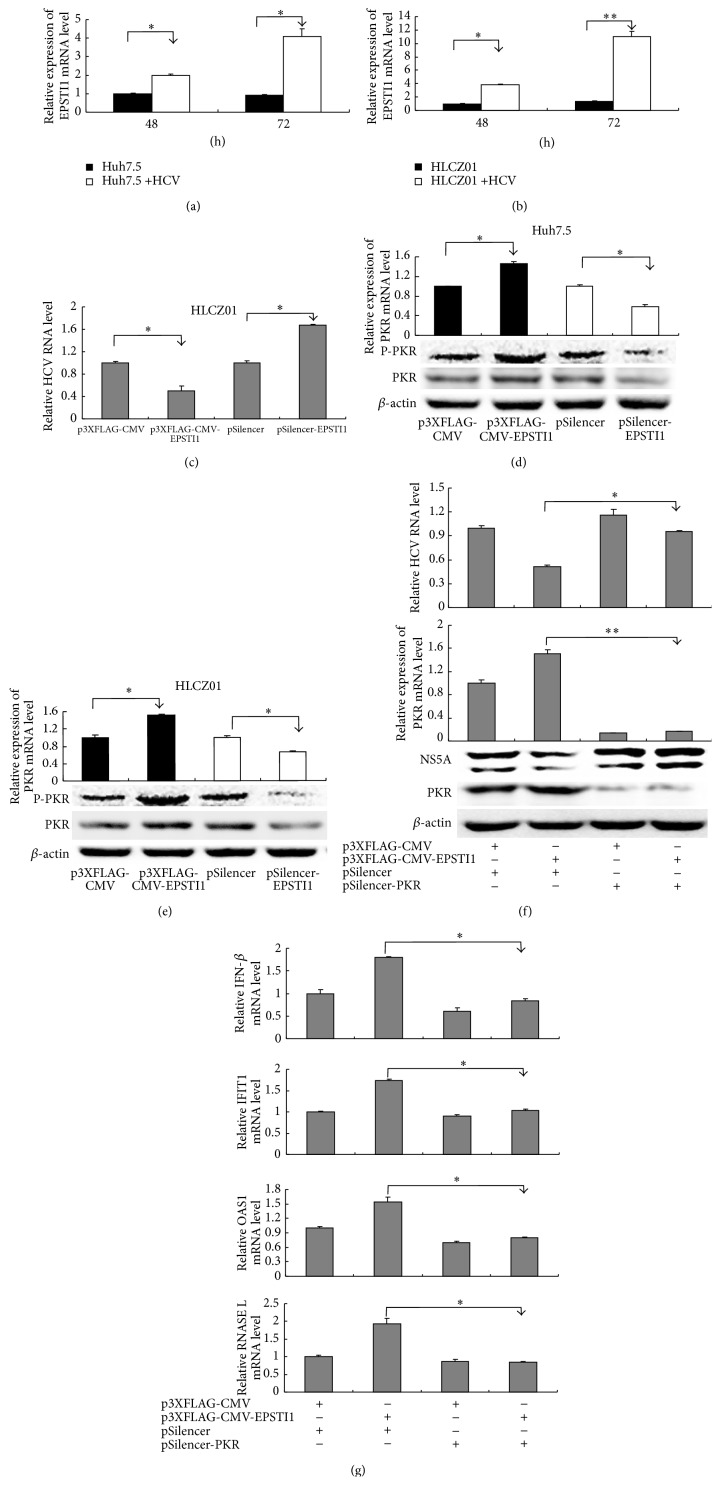
The inhibition of HCV triggered by EPSTI1 is dependent on PKR. ((a) and (b)) Huh7.5 cells (a) and HLCZ01 cells (b) were infected with HCV (MOI = 0.1). EPSTI1 mRNA was detected with real-time PCR and normalized with GAPDH. ((c), (d), and (e)) Huh7.5 and HLCZ01 cells were transfected with p3XFLAG-CMV-EPSTI1 or pSilencer-EPSTI1 followed by HCV infection. Intracellular HCV RNA and PKR mRNA were detected by real-time PCR and normalized with GAPDH. The proteins of PKR and p-PKR were detected by western blot. ((f) and (g)) p3XFLAG-CMV-EPSTI1 or/and pSilencer-PKR was delivered into HLCZ01 cells followed by HCV infection. (f) HCV RNA and PKR mRNA were detected by real-time PCR and normalized with GAPDH. NS5A and PKR proteins were detected by western blot. (g) IFN-*β*, IFIT1, OAS1, and RNASE L mRNA were detected by real-time PCR and normalized with GAPDH.

In the many antiviral mechanisms of clarified-ISGs, positive regulation of innate response signaling by ISGs is a key way to clear virus. To investigate whether EPSTI1 employs this mechanism, we overexpressed EPSTI1 in HCV-infected or noninfected cells and detected the expression of some known antiviral genes including IFI44L, ISG12a, 1-8U, ISG60, PKR, and OAS1. Only PKR and OAS1 were upregulated under this condition compared to the mock overexpression (Figure 5). Interestingly, PKR and OAS1 mRNA in cells with HCV infection showed higher expression levels than that in mock-infected cells (Figures 5(e) and 5(f), lane 3 compared with lane 1 and lane 4 compared with lane 2). Moreover, the level of EPSTI1 mRNA increased in both HCV-infected Huh7.5 cells and HCV-infected HLCZ01 cells in comparison with naïve cells (Figures 6(a) and 6(b)). The inhibition of HCV infection by EPSTI1 was also confirmed in the HLCZ01 cells (Figure 6(c)).

Next, we want to investigate whether EPSTI1 regulates the expression of PKR and OAS1 during HCV infection. Strikingly, the level of PKR, especially phosphorylated PKR, was increased or reduced when we overexpressed or knocked down EPSTI1 in HCV-infected cells, respectively (Figures 6(d) and 6(e)). To further determine the role of PKR in EPSTI1-mediated anti-HCV process, we knocked down PKR in the EPSTI1-overexpressed cells (Figure 6(f)). Silencing of PKR reversed the suppression of HCV as well as the induction of PKR by overexpression of EPSTI1 (Figure 6(f)), suggesting that PKR play a vital role in EPSTI1-mediated antiviral activity. Recent study revealed that PKR is involved in antiviral innate immune response by induction of IFN-*β* and a subset of early ISGs during HCV infection. In our study, we found that IFN-*β* and two early ISGs (OAS1 and IFIT1) were upregulated in the EPSTI1-overexpressed cells and this effect was attenuated when we simultaneously silenced PKR in the EPSTI1-overexpressed cells (Figure 6(g)). OAS1 is a known antiviral ISG and initiates viral RNA degradation through the RNase L pathway. The level of RNase L mRNA was also enhanced in the EPSTI1-overexpressed cells and this process was reversed by PKR knockdown (Figure 6(g)). These data indicated that EPSTI1 exhibits anti-HCV activity through PKR pathway.

### 3.6. EPSTI1 Increases PKR Expression by Activating PKR Promoter

**Figure 7:**
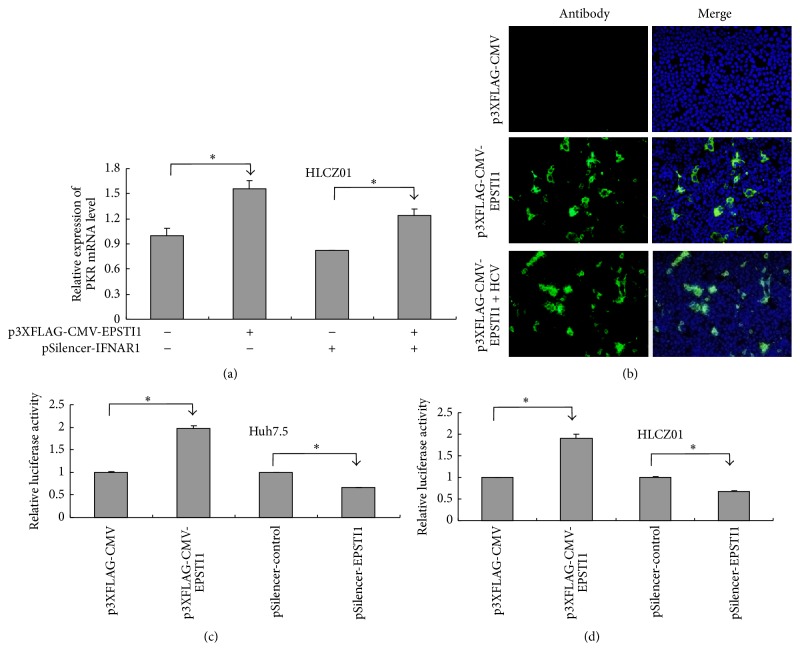
EPSTI1 upregulates PKR expression by activating PKR promoter. (a) HLCZ01 cells were transfected with p3XFLAG-CMV-EPSTI1 or/and pSilencer-IFNAR1 followed by HCV infection (MOI = 0.1). PKR mRNA was detected by real-time PCR and normalized with GAPDH. (b) Huh7.5 cells were transfected with p3XFLAG-CMV-EPSTI1 followed by HCV infection. The cells were assayed by immunofluorescence at 48 h postinfection. ((c) and (d)) Huh7.5 (c) and HLCZ01 cells (d) were cotransfected with pGL3-PKR promoter and p3XFLAG-CMV-EPSTI1 or pSilencer-EPSTI1 followed by HCV infection. Cell extracts were made at 24 h postinfection followed by luciferase determination.

During viral infection, PKR could be induced by interferon response pathway. To determine whether the expression of PKR is induced directly by EPSTI1, we silenced IFNAR1 using shRNA in HCV-infected cells. The effects of IFNAR1 knockdown by shRNA were confirmed (Figures S3A and S3B). Loss function of IFNAR1 did not reverse the induction of PKR mRNA by EPSTI1 overexpression, suggesting that EPSTI1 may regulate PKR expression directly and this regulation is not dependent on type I interferon response pathway (Figure 7(a)). Recent study showed that EPSTI1 is a nucleocytoplasmic protein [20]. In our study, we found that HCV infection can promote EPSTI1 protein moving from cytoplasm to nucleus (Figure 7(b)). Therefore, we hypothesized that EPSTI1 may act as a transcription factor to regulate the activity of PKR promoter. To verify this possibility, we cotransfected p3XFLAG-CMV-EPSTI1 or pSilencer-EPSTI1 with pGL3-PKR promoter in the HCV-infected cells and found that EPSTI1 overexpression can activate PKR promoter and silencing of EPSTI1 can attenuate the activity of PKR promoter (Figures 7(c) and 7(d)). These data indicated that EPSTI1 induces PKR expression through activating PKR promoter.

## 4. Discussion

IFNs are cytokines produced and released by host cells in response to the presence of pathogens such as viruses, bacteria, and parasites. IL-28A signals through a heterodimer which comprises the interleukin-28 receptor chain (IL-28R*α*) and the interleukin-10 receptor *β* chain (IL-10R*β*). The distribution of IFN-*α* receptor and even IL-10R*β* is found on a wide variety of cell types, while IL-28R*α* is found primarily on epithelial cells. The clinical trial of IFN-*λ* holds expected benefit that it may result in fewer adverse effects than IFN-*α* treatment. Recently, many studies revealed that the IL-28B genetic variations are associated with HCV clearance or response to pegylated IFN-*α* plus ribavirin treatment [6–10] and indicated that IFN-*λ* may play a critical role in innate defense system. Previous works have shown that IL-28A inhibits HCV replication in HCV replicon cells [12, 13]. In this study, we investigated the antiviral effect of IL-28A on the entire life cycle of HCV in the infectious cell culture system and elucidated the mechanism of how EPSTI1, an IL-28A-induced ISG, inhibits HCV replication.

Although the inhibitory effect of HCV RNA replication by IL-28A was reported in the previous study, whether IL-28A affects other steps of HCV life cycle is still unknown. We systematically analyzed the biological function of IL-28A using infectious cell culture system. In our study, we verified our previous results and found that IL-28A inhibits HCV replication in both HCV full-length replicon cells and viral-infected hepatocytes (Figure 1). Importantly, IL-28A suppresses the production of infectious viral particles (Figure 2). However, the entry of HCV is not affected by IL-28A treatment (Figure 1(f)).

We assessed whether IL-28A and IFN-*α* combination could produce synergistic effects on antiviral activity in the infectious cell culture system. Our data showed that the IL-28A and IFN-*α* do synergistically inhibit HCV replication (Figure 3(a)). Moreover, we detected the expression of a series of ISGs induced by IL-28A and IFN-*α* alone or in combination. Our results revealed that the peak gene expression induced by IFN-*α* occurs earlier than that induced by IL-28A, while the induction of ISGs by IL-28A lasts longer than IFN-*α* (Figure 3(b)). It was reported that IFN-*λ* prolongs the duration of activated STAT1 and the expression of STAT1 in comparison with IFN-*α* [26]. The unphosphorylated STAT1 is implicated in the prolonged increase of phosphorylated STAT1 by IFN-beta or IFN-gamma, which promotes the expression of IFN-induced immune regulatory genes [30, 31]. Whether IL-28A and IFN-*α* employ this mechanism is needed to be clarified. Although the induction of ISGs differs between IL-28A and IFN-*α*, the combination of two IFNs synergistically enhances the expression of ISGs (Figure 3(b)).

EPSTI1 belongs to the set of ISGs. There was a report that EPSTI1 is upregulated in hepatitis E virus-infected patients compared to normal individuals and the expression level of EPSTI1 is associated with the persistence of HEV infection [32]. In our study, EPSTI1 is induced more markedly by IL-28A than IFN-*α* (Figure 3(b)). Overexpression or knockdown of EPSTI1 significantly enhances or impairs the antiviral effect of IL-28A (Figures 4(a)–4(c)). Interestingly, EPSTI1 is induced by HCV infection and overexpression of EPSTI1 alone inhibits viral replication (Figures 4 and 6). PKR can sense HCV dsRNA to activate a kinase-independent signal transduction cascade, which drives the induction of specific ISGs and IFN-*β* production to restrict virus survival [33]. OAS1 also can recognize viral dsRNA, which activates OAS1 and initiates RNA degradation within the infected cell via the RNase L pathway to clear virus [34]. Here, we found that the expression of PKR, OAS1, and RNase L is regulated by EPSTI1 (Figures 5 and 6), providing a novel mechanism by which EPSTI1 mediates the anti-HCV activity.

There were two hepatoma cell lines, Huh7.5 and HLCZ01, that were used for investigating the mechanisms of IL-28A and EPSTI1 to inhibit HCV propagation in our study. Huh7.5 is a highly HCV permissive cell line derived from the human hepatoma cell line Huh7 [35]. However, a mutation (T55I) within the RIG-I gene was found in Huh7.5 cells, which leads to the inactivation of RIG-I-dependent pathway [36]. In contrast with Huh7.5, HLCZ01 cell line mounts an intact antiviral immune response, including induction of type I IFN and ISGs, during HCV infection [21, 37]. In this study, we found that forced expression of EPSTI1 exhibits HCV suppression in both Huh7.5 and HLCZ01 cells (Figures 4 and 6), indicating that the antiviral action of EPSTI1 possibly does not depend on RIG-I pathway.

The advances in functional data analysis facilitate the identification of more unknown antiviral ISGs and reveal their mechanisms of action with respect to the viral life cycle and host cell processes. With hundreds of genes induced by IFNs, it is reasonable to assume that any step of the viral life cycle could be targeted for inhibition. However, IL-28A exhibits no inhibition to HCV entry in our study.

**Figure 8:**
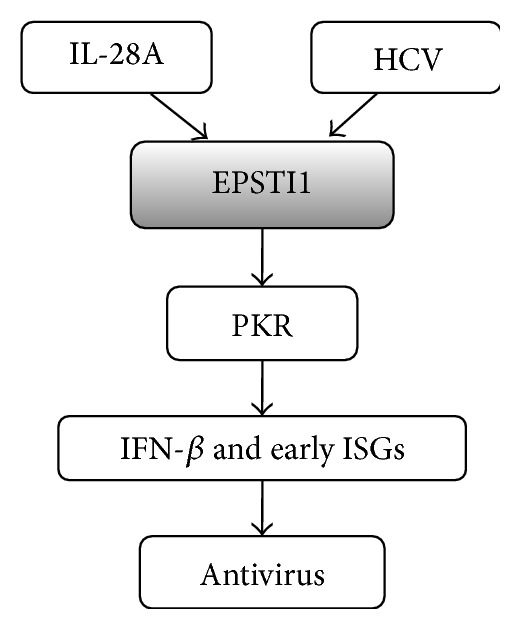
Model for EPSTI1-mediated anti-HCV activity. EPSTI1 induced by IL-28A and HCV infection can activate PKR expression, which leads to the induction of PKR-dependent genes, including IFN-*β* and some early ISGs, to restrict HCV infection.

In summary, our results demonstrated that IL-28A effectively suppresses HCV replication, assembly, and release. IL-28A and IFN-*α* synergistically inhibit HCV replication, though they induce the different profiles of ISGs not only in the magnitude but also in the time course. EPSTI1, one of ISGs, can be induced by HCV infection and play a pivotal role in IL-28A-mediated anti-HCV activity. EPSTI1 exhibits direct anti-HCV activity through upregulation of PKR and PKR-dependent early ISGs in the HCV-infected cells (Figure 8). Recently, a study revealed that EPSTI1 interacts with the valosin-containing protein (VCP) in cytoplasm, resulting in the degradation of I*κ*B*α* and subsequent activation of NF-*κ*B in the nucleus [38]. Our results revealed that HCV can promote EPSTI1 protein moving from cytoplasm to nucleus. Forced expression of EPSTI1 activates PKR promoter. These data suggested that EPSTI1 modulates PKR expression through activating PKR promoter. In conclusion, our study demonstrated that EPSTI1 acts as a novel anti-HCV ISG and plays an essential role in IL-28A-mediated antiviral activity, which provides a promising target for the development of therapeutic drugs for HCV infection.

## Supplementary Material

Supplementary Figure 1: presents the inhibitory percentages of IL-28A to suppress HCV life cycle at 48h and 72h.
Supplementary Figure 2: presents the EC50 of IFN-α to inhibit HCV RNA replication in HCV-infected Huh7.5 cells.
Supplementary Figure 3: presents the silencing effect of IFNAR1 shRNA in the HLCZ01 cells.
