# Supplementary material for: EPSTI1 Is Involved in IL-28A-Mediated Inhibition of HCV Infection
Source: Mediators Inflamm. 2015 Jun 3;2015:716315. doi: 10.1155/2015/716315 (PMC4469844; doi:10.1155/2015/716315)
Supplement: Supplementary file 1 — Supplementary Figure 1: presents the inhibitory percentages of IL-28A to suppress HCV life cycle at 48h and 72h. Supplementary Figure 2: presents the EC50 of IFN-α to inhibit HCV RNA replication in HCV-infected Huh7.5 cells. Supplementary Figure 3: presents the silencing effect of IFNAR1 shRNA in the HLCZ01 cells. [file 716315.f1.pdf]

SUPPLEMENTARY FIGURES

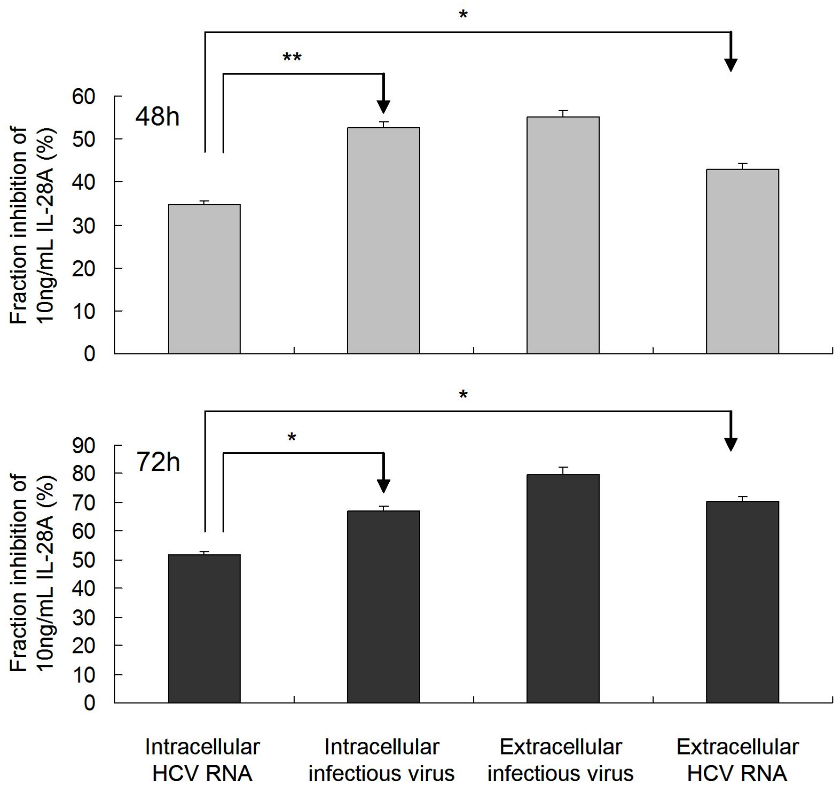

Fig. S1. The inhibitory percentages of IL-28A. Huh7.5 cells were infected with HCV (MOI=0.1) for 6h at 37℃. The medium was then removed and the cells were incubated with10ng/mL IL-28A for 48h (upper) or 72h (lower). Intracellular and extracellular HCV RNA was detected by real-time PCR. Intracellular and extracellular infectious virus was detected by FFU (focus-forming unit) assay on naive Huh7.5 cells.

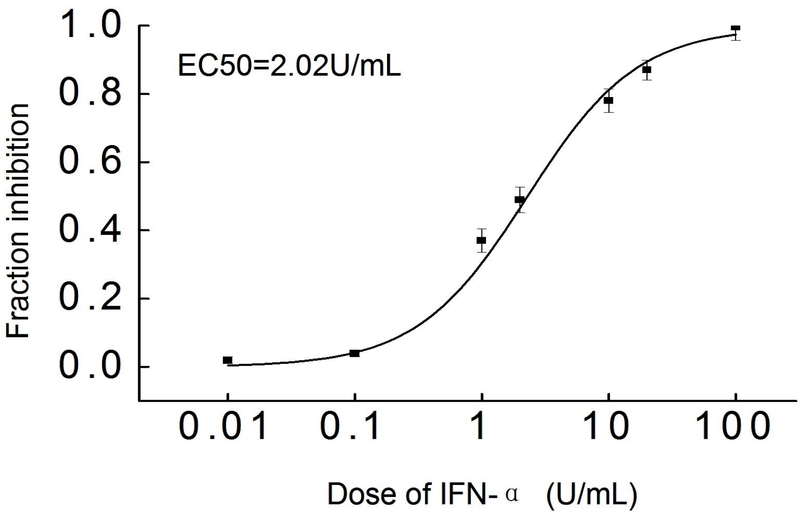

Fig. S2. The EC50 of IFN-α for inhibition of HCV RNA replication. Huh7.5 cells were infected with HCV (MOI=0.1) for 6h at 37℃. The medium was then removed and the cells were incubated with different doses of IFN-α for 72h. Intracellular viral RNA was detected by real-time PCR. The results are the average of three independent experiments performed in triplicate.

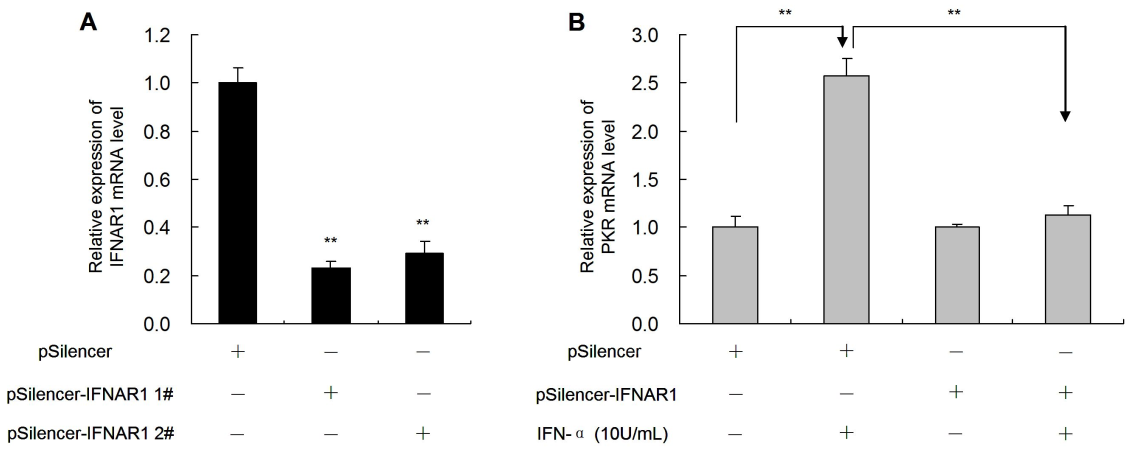

Fig. S3. The effect of IFNAR1 knockdown. (A) HLCZ01 cells were tranfected with pSilencer vector or pSilencer-IFNAR1 1# or 2# for 48h. The expression of IFNAR1 was

measured by real-time PCR. (B) The cells were treated as described in part A followed by IFN-α (10U/mL) treatment for 12h. The expression of PKR was detected by real-time PCR.
